# Sequence of Molecular Events in the Development of Alzheimer’s Disease: Cascade Interactions from Beta-Amyloid to Other Involved Proteins

**DOI:** 10.3390/cells13151293

**Published:** 2024-07-31

**Authors:** Soghra Bagheri, Ali Akbar Saboury, Luciano Saso

**Affiliations:** 1 Medical Biology Research Center, Health Technology Institute, Kermanshah University of Medical Sciences, Kermanshah 6714415185, Iran; 2 Institute of Biochemistry and Biophysics, University of Tehran, Tehran 1417614335, Iran; saboury@ut.ac.ir; 3 Department of Physiology and Pharmacology “Vittorio Erspamer”, Sapienza University, 00185 Rome, Italy; luciano.saso@uniroma1.it

**Keywords:** APOEɛ4, TREM2, NMDA, women, cholesterol, AD mechanism

## Abstract

Alzheimer’s disease is the primary neurodegenerative disease affecting the elderly population. Despite the first description of its pathology over a century ago, its precise cause and molecular mechanism remain unknown. Numerous factors, including beta-amyloid, tau protein, the APOEε4 gene, and different metals, have been extensively investigated in relation to this disease. However, none of them have been proven to have a decisive causal relationship. Furthermore, no single theory has successfully integrated these puzzle pieces thus far. In this review article, we propose the most probable molecular mechanism for AD, which clearly shows the relationship between the main aspects of the disease, and addresses fundamental questions such as: Why is aging the major risk factor for the disease? Are amyloid plaques and tau tangles the causes or consequences of AD? Why are the distributions of senile plaques and tau tangles in the brain different and independent of each other? Why is the APOEε4 gene a risk factor for AD? Finally, why is the disease more prevalent in women?

## 1. Introduction

Alzheimer’s disease (AD) is the most common neurodegenerative disorder associated with aging. It was estimated that there were approximately 50 million people with AD worldwide in 2019, and this number is projected to exceed 152 million by 2050. About USD 1 trillion was spent on this disease in 2019 alone [1]. Despite the fatality of AD and its high prevalence, as well as the later economic burden, no effective treatment has been found for it more than a hundred years after its discovery, because the mechanism of its occurrence has not yet been discovered [2]. Numerous review articles have been written on different aspects of AD and its mechanism, yet none have offered a comprehensive depiction of the interrelation between the various elements of this enigma. As mentioned in the abstract, what sets this article apart from others is that it proposes a mechanism that not only illustrates the relationship between key aspects of the disease but also answers critical questions about the disease.

## 2. Major Pathological Hallmarks

### 2.1. Neurofibrillary Tangles

The main hallmarks of AD include beta-amyloid (Aβ) plaques and neurofibrillary tangles (NFTs). Evidence indicates that NFTs typically begin to form in almost all individuals at around 60–70 and tend to increase with advancing age [3,4,5]. It is speculated that neurons may even live for decades despite having NFTs [6]. The Braak NFT staging shows that dementia does not occur without the threshold level of tangles [7]. Based on the data presented in Table 2 of reference [7], it was found that individuals aged between 47 and 96 who showed Braak stage 0, I, or II did not show signs of dementia, regardless of Aβ deposition. The critical point for the brain is when tangles reach level III. Individuals with Braak stage III or IV are considered to be at a higher risk of developing AD. For a thorough examination of Braak staging, we recommend referring to various articles, including [8,9,10].

According to Braak’s table, it is important to note that individuals with Braak stages V and VI are linked to high-level Aβ deposition (stage C: end-stage deposits) or dementia [7]. This does not necessarily mean that all individuals with Braak stage V and VI will develop dementia, but it does suggest that those with dementia will experience an increase in tangle levels to that extent. Evidence suggests that amyloid plaques promote tau phosphorylation and tangle formation [3,11,12], and individuals in these two groups in Braak’s table exhibit the highest Aβ deposition.

After the formation of the blood–brain barrier (BBB), the microglia population does not proliferate from the blood but undergoes self-regeneration [13]. Telomere shortening in microglia leads to their aging [14], as evidenced in AD [15]. In aged microglia, phagocytic strength decreases [16]. Indeed, the microglia phenotype undergoes alterations as individuals age, leading to a decrease in the expression of various proteins that interact with amyloid, such as CD36 and TREM2 [16,17]. On the other hand, Aβ is produced in the brain during its normal functioning [18,19] and is eliminated by microglia [20]; however, as microglia age and their ability to clear Aβ diminishes, the accumulation of Aβ in the brain increases. Unremoved excess Aβs can form dimers, which can create channels in neuronal membranes, leading to an influx of excessive calcium into nerve cells [21,22]. Calcium induces tau phosphorylation by acting on intracellular proteins [23,24,25,26]. This phosphorylation results in the detachment of tau from the microtubule and the accumulation of tau. Subsequently, hyperphosphorylated tau leads to NFT formation [23].

Unlike plaques, tangles follow a specific pattern of development and increase over time [3,5,7]. The mechanism underlying tangle formation in the brain follows a distinct pattern, which is different from plaque formation. Different brain regions exhibit varying levels of activity [18,19]; the initiation of tangle formation occurs in regions that show high levels of activity. As microglia age, tangles begin to appear in other brain regions over time. Furthermore, the connection between Aβ and synapse activity has been established in numerous studies. Aβ42 reduction has also been shown to reduce synapse transmission by up to 50% [27,28].

### 2.2. Senile Plaques

Another indication of AD is the presence of Aβ plaques. Individuals diagnosed with AD, as well as some individuals who are healthy, exhibit Aβ plaques [3], indicating that plaque formation is a necessary but not sufficient factor in the development of AD. Therefore, it is crucial to consider why and how these plaques are formed when examining the occurrence of AD. Alongside the formation of tangles, two other natural processes that take place during aging are immunosenescence [29] and an increase in the permeability of the BBB [30]. Generally, infectious agents are prevented from entering the brain by the BBB, although there have been instances where infectious agents have managed to breach the BBB and reach the brain [31,32,33]. The prevalence of infections among the elderly is significant, with one-third of elderly deaths attributed to infections [34]. In addition to the increased susceptibility to infection at this stage in life, an imperfection in the BBB is also a significant factor in allowing microbes to enter the brain [35,36]. Should an infectious agent breach the brain, microglia serve as the primary line of defense against it. Microglia are brain-specific macrophages that engulf foreign particles and halt the spread of infectious agents [37]. The synaptic space experiences elevated levels of APP and Aβ as a reaction to the invasion of pathogens in the brain [27,28]. Aβs (like other antimicrobial peptides [38,39]) can encapsulate the infectious agent or create pores in the microbial membrane [22,40,41], ultimately leading to the microbe’s demise. The microbe’s debris is eliminated by the microglia [42,43]. However, in elderly individuals whose microglia are incapable of phagocytosis, these remnants are not cleared, resulting in the formation of diffuse plaques in the brain.

It is noteworthy that certain regions of the brain, such as the hippocampus, contain high levels of copper at the synapse [44]. When pathogens invade these areas, they are met with various defense mechanisms of the brain. Apart from copper, which acts as an antimicrobial agent [45], the uptake of copper by microglia serves as another line of defense [46,47,48]. Furthermore, copper uptake from the synapse leads to copper deficiency in neurons, leading to increased dimerization of APP and activation of the gamma-secretase enzyme, both of which contribute to increased Aβ production (antimicrobial peptide) [49,50]. Additionally, the presence of copper, along with the active microglia in the synapse, causes the microglia to become hyperactive, leading them to avoid Aβ phagocytosis and instead produce inflammatory cytokines [51,52,53]. Since Aβ is antimicrobial [27], the accumulation of amyloid seems to be a defense mechanism of the brain against infectious agents.

**Figure 1:**
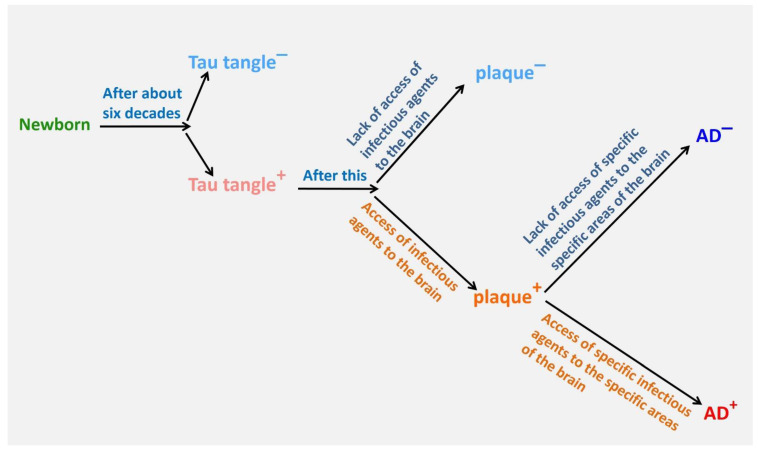
Sequential events in the development of Alzheimer’s disease.

**Figure 2:**
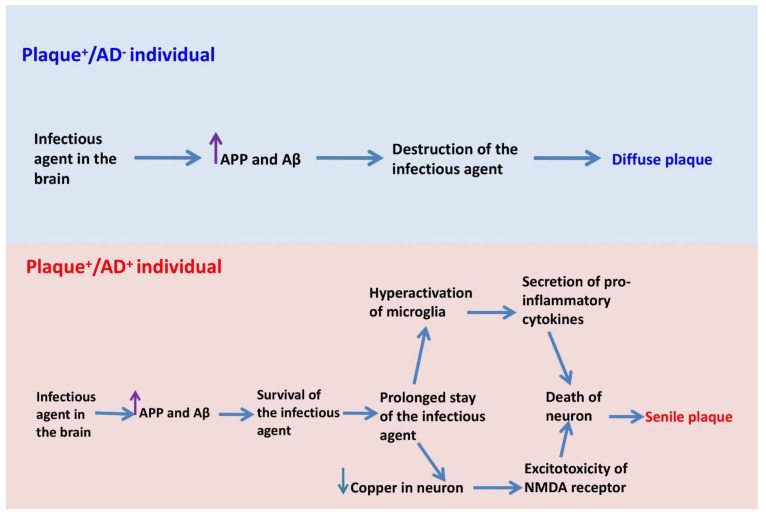
Differences in the formation of diffuse plaques and senile plaques in Alzheimer’s disease.

Some pathogens exhibit resistance to Aβ or copper [54,55] and are not eliminated by a simple defense. In such instances, the brain is capable of eliminating them with a more robust defense mechanism over an extended period [56]. The critical and vulnerable point lies in the infiltration of these infectious agents into the brain region that utilizes high levels of copper in the synaptic space. On the one hand, microglia, in response to the presence of pathogens, heighten excitotoxicity by generating inflammatory cytokines and increasing glutamate production [57,58]. On the other hand, microglia remove copper from the synapse, resulting in neurons becoming copper-deficient. Prolonged exposure to these conditions (due to pathogen resistance) can lead to excitotoxicity of NMDA receptors [59]. Calcium release into neurons can lead to further tangles [60,61,62,63] or even neuronal death. Consequently, in elderly individuals whose microglia are unable to phagocytose neuronal debris, senile plaques develop. Reports on the dense plaques containing proteins and components of neurons [64] support this assertion. Furthermore, the presence of tau-containing plaques, which have been demonstrated to be a major cause of memory impairment [65,66,67], confirms the existence of this mechanism (Figure 1 and Figure 2).

There is substantial evidence indicating the presence of germs in the brains of AD patients [68,69]. In addition to breaching the BBB for various reasons, germs can also infiltrate the brain through olfactory receptors. Olfactory impairment is often the initial sign of AD, attributed to entorhinal involvement [70]. Some pathogens do not interact with the BBB to enter the brain [71], and instead, due to the unique structure of olfactory receptors [72], they enter the brain in this manner. Evidence suggests that olfactory problems occur a decade before the onset of AD symptoms [73]. Indeed, the olfactory is impaired when tangles are formed in the entorhinal [70]. Braak’s findings also indicate that the entorhinal region is one of the first areas affected by tangle [7]. The presence of pathogens such as HSV1, HSV2 [74], and γHV [75] in plaques can be evidence of their entry into the brain through the olfactory system and damage to the entorhinal region. Chlamydia pneumoniae, closely linked to AD [76], has been found to enter the brain through respiration [77]. Decreased cognition in patients with serum HSV^+^ and improved symptoms with antiviral drugs are further evidence of the presence of the virus in the brain and its impact on cognitive functioning [74]. Additionally, other studies have found that HSV^+^ individuals were more likely to develop AD [78,79,80].

## 3. What Does the Specific Threshold of Tangles Show?

Previous sections have highlighted the fact that it takes 6–7 decades for tangles to reach the threshold. However, the reason behind tangle levels reaching the threshold at this age and the necessity of having a specific level of them to cause AD remains a question. The physiological (biological) age of individuals with the same chronological age can differ, as aging may occur earlier or later than the chronological age due to genetic and environmental factors. Apart from these cases, in general, in a brain that is almost seventy years old, the microglia are also seventy years old.

Indeed, the development of plaque in older individuals and its absence in younger individuals is directly linked to the aging process of microglia. In young people, microglia effectively eliminate germs that enter the brain, but as microglia age to a certain point (when tangles reach Braak stage III) their ability to clear external factors diminishes. At this stage, the senescence of microglia leads to a significant reduction in their phagocytic capacity, resulting in the formation of plaque, as the killed germs are not effectively removed.

If the germs manage to enter the brain (when the tangles have reached at least level III) and are resistant, and they reach the hippocampus, this can lead to the removal of copper from the synapse and the hyperactivity of the microglia occurs due to the prolonged presence of the germs. These conditions can result in the death of neurons, which have a large amount of Aβ deposited inside and outside. This accumulation remains as a plaque that contains other components in addition to Aβ. Furthermore, in the advanced stages of AD, when the microglia are older, senile plaques can form in any area of the brain.

Several researches provide evidence for this theory; some studies have shown the correlation between aging microglia and neurofibrillary degeneration. Some studies have indicated that the aging of microglia is prevalent in Braak stages V and VI and is closely associated with neurofibrillary degeneration [81]. Another study showed the spread of microglia and tau aggregates in a Braak-like manner within the same spatial areas [82]. Additionally, further research suggests that the microglia phenotype varies between the early stages (Braak 2) and late stages (Braak 4) of the disease [83]. In another study, it was noted that microglia in Braak II samples displayed a healthy morphology characterized by extensively ramified processes. Conversely, microglial cells in Braak V–VI samples exhibited shortened and less branched processes that were often deformed, showing cytoplasmic abnormalities and even fragmentation [84]. Aged microglia have previously been shown to be associated with tau pathology. Furthermore, the same study using Braak staging of AD neuropathology has revealed that microglial senescence (dystrophy) occurs prior to the spread of tau pathology [85].

## 4. Spread Patterns of Plaques and Tangles in Alzheimer’s Disease

Plaques in the human brain do not exhibit a specific pattern in terms of distribution, number, size, or composition, unlike tangles. Research has shown that during the pre-AD stage, plaques and tangles are located in different regions [86]. The hypothesis proposed in this study suggests that as microglia age, tangle formation initiates in the more active brain regions and gradually expands in both distribution and quantity following a certain pattern. Conversely, plaque formation, besides microglia senescence, is influenced by the introduction of an infectious agent, which varies in timing, extent, and type of infection for each individual.

APP mutations in early-onset AD patients lead to both tau and plaque pathology, whereas tau mutations only result in tau pathology [27]. Interestingly, plaque formation seems to occur independently of the presence of tangles at the plaque formation site, as indicated by many studies. Some reports suggest that cortical plaques occur before tau pathology [60]. According to the hypothesis proposed in this study, the development of tau pathology and tangle formation, after reaching a specific level of microglia aging, is influenced by brain activity (Aβ production and clearance rate). Regions of the brain with low Aβ production may not display tau pathology despite microglial senescence. However, if pathogens invade these areas, the likelihood of plaque formation is high due to the age-related decline in microglial phagocytic ability.

## 5. Molecular Events Leading to Tangle Formation

Multiple studies on AD mouse models have demonstrated a direct relationship between Aβ42 and tau phosphorylation [61,62,63]. This association has also been validated in recent research [87]. Other studies have indicated that Aβ42 levels in cerebrospinal fluid (CSF) decrease several years before the onset of dementia symptoms [88]. A reduction in Aβ42 in CSF suggests an increase in the brain [88,89]. Furthermore, an elevation in the Aβ42/Aβ40 ratio has been observed in both familial and late-onset AD [90]. APP [91,92] and certain Presenilin-1 (PSEN1) mutations [91] result in an increase in the Aβ42/Aβ40 ratio and tau phosphorylation in early-onset AD.

Several investigations on mouse models of AD have revealed that tau phosphorylation is regulated by calcium, and cognition decreases with tau hyperphosphorylation [93]. Moreover, research conducted on cultured neurons has indicated that calcium plays a role in mediating tau phosphorylation [24,25]. Additionally, alterations in calcium levels have been shown to impact the metabolism of APP and tau, as observed in AD [24]. Indeed, numerous protein kinases and phosphatases in neurons rely on calcium, and elevating the level of cytosolic calcium through their modulation results in tau hyperphosphorylation [24,94]. For instance, increasing calcium levels leads to enhanced microsomal prostaglandin E synthase 1 (mPGES1) activity, resulting in the synthesis of prostaglandin E2. Subsequently, prostaglandin E2 triggers the activation of the calcium-sensitive cysteine protease, calpain, via EP1, EP2, and EP3 receptors. Upon calpain activation, the p35 protein undergoes cleavage to generate its truncated form, p25. This p25 fragment then activates CDK5, ultimately leading to tau phosphorylation [93]. It has been reported that the overexpression of calpain, which regulates the activity of AD-related proteins such as tau kinase, occurs from Braak stage III onwards [26].

Numerous reports have shown that the accumulation of Aβ not only impacts various membrane receptors involved in calcium uptake but can also directly channel in the membrane and increase intracellular calcium [95,96]. It has been established that the presence of a dimer is essential for the creation of a channel, and studies indicate that Aβ dimers regulate tau phosphorylation [97]. Interestingly, studies have demonstrated that Aβ42 dimers exhibit distinct characteristics compared to Aβ40 dimers [98]. Specifically, Aβ42 dimers have been found to adhere to microbial membranes and possess to eliminate them, whereas Aβ40 dimers are significantly less effective [98]. Furthermore, other studies have shown that monkeys with plaques in their brains, but lacking dimers, do not exhibit signs of dementia [99]. This finding supports the notion that plaque formation does not directly result in neuronal destruction; rather, it is the calcium penetration and tangle formation that lead to neuronal death.

Although amyloid plaques are found in various parts of the brain, AD typically originates in the hippocampus, which is the area most affected by damage throughout the progression of the illness. Indeed, apart from the regulation of NMDA receptors by copper, various factors contribute to the vulnerability of this region to neuronal damage. These include its proximity to the entorhinal (which is more easily accessible by infectious agents compared to other parts of the brain), as well as its high activity, which accelerates and intensifies tangle formation and the subsequent damage to the hippocampus. Senile plaques have been shown to reduce cognition in individuals without dementia, while diffuse plaques are ineffective [67]. The presence of senile plaques in non-demented individuals has been documented in several studies [100]. In fact, senile plaques occur when neurons are destroyed (according to the present hypothesis), while during the formation of diffuse plaque, neurons are still intact. For this reason, the presence of senile plaque is associated with cognitive deficits, and undoubtedly, the effect of senile plaque on cognition depends on the place of its occurrence. Typically, in a 70-year-old’s brain, when tangles exceed a certain level and a resistant infectious agent infiltrates the brain areas that utilize copper to block the NMDA receptors, senile plaques develop, leading to the onset of AD symptoms.

## 6. Explaining the Roles of Different AD Risk Factors by the Present Hypothesis

The accuracy of the hypothesis presented in this study has been supported by numerous studies; for example, the investigation conducted in citation [3]. According to this study, older individuals without dementia but with plaque have plaques in the cerebral cortex, not in the entorhinal or hippocampal regions. The most important case is an individual who has not suffered from dementia despite having numerous tangles and plaques. The pathological details of this person are important. Firstly, his/her plaques were mostly primary plaques (i.e., the neurons were not destroyed). The presence of different plaques in healthy individuals and AD patients has also been documented in other studies [101,102]. Secondly, the plaques were mainly in the neocortex (meaning the neurons were not destroyed because they were outside the brain’s sensitive areas for germs to enter). Thirdly, there were mature plaques in the hippocampus (meaning the entry of an infectious agent into the hippocampus caused neuronal death and senile plaque formation). Another key point of this study is that the distribution and severity of tangles and plaques are not the same, confirming that the plaque formation is not a function of tangle formation; instead, their formation is a function of accidental events. Another issue is that the distributions of primary and mature plaques are different and do not have a one-to-one conversion [3]. This supports the two different pathways proposed in the current study for the formation of diffuse and senile (dense) plaques. In fact, there is ample evidence that the composition of diffuse and dense plaques differs. More Aβ42 and Aβ43 [103], neuron nuclei [104], and neuron proteins [105] have been reported in dense plaques.

According to the hypothesis outlined in this study, the relationship between different risk factors and AD can be effectively elucidated. Following aging, the most important risk factor for AD is APOEε4. APOE comes in three variants, with APOEε3 being the standard, while APOEε2 and APOEε4, respectively, decrease and increase the risk of AD [27]. In the United States, it has been observed that half to two-thirds of AD patients possess APOEε4 [106,107]. APOEε4 has been demonstrated to lower the age at which AD develops [108], indicating that APOEε4 leads to the premature formation of tangles, similar to what occurs in Down syndrome and early-onset AD. APOEε4 has been demonstrated to decrease neurons’ cholesterol [109]. Conversely, research has indicated that a decrease in cholesterol within neurons leads to an elevation in tau phosphorylation [110,111].

**Figure 3:**
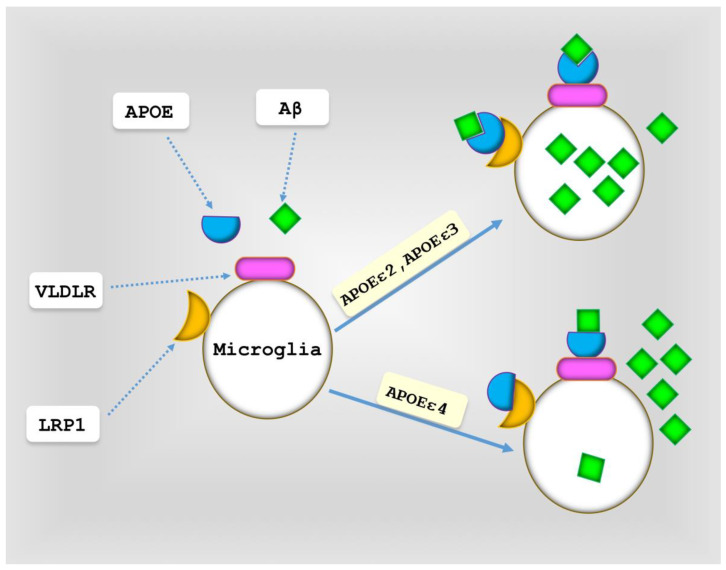
Aβ clearance by APOE isoforms. APOEε4 hinders the clearance of Aβ by attaching to LRP1 and VLDLR, whereas APOEε2 and APOEε3 facilitate the uptake of Aβ by binding to LRP1.

Aβ and cholesterol are taken up by microglia when they are bound with APOE [112], with TREM2 being for these complexes [113,114]. Studies indicate that APOEε4 isoform hinders Aβ clearance in a mouse model [27,109]. Upregulation of APOEε4 has been reported in the microglia of AD patients. Some findings have shown that APOEε4 inhibits Aβ clearance by binding to LRP1 and VLDLR (both of which are receptors involved in Aβ clearance) [27,115]. Conversely, APOEε2 and APOEε3 promote Aβ uptake by binding to LRP1 [115] (Figure 3).

In healthy individuals under the age of 70, those with APOEε4 homozygosity have been found to have the lowest Aβ levels in CSF (indicating the highest Aβ deposition in the brain) [89]. Moreover, it has been demonstrated that APOEε4 aggregates with Aβ more rapidly than other APOE variants [116] and colocalizes at the synapse with Aβ oligomers [117]. Evidence suggests that APOEε4 and Aβ have a synergistic effect on increasing the tau load [118], indicating that they not only have individual effects on increasing the tau load but are also interrelated. Some evidence has indicated that APOEε4 increases Aβ load in addition to tau load [119]. It has been observed that in individuals who are Aβ^+^ (showing amyloid deposition), APOEε4 is strongly associated with tangles, while in Aβ^-^ individuals, it has no association with tangles [120]. In general, it can be concluded that APOEε4 contributes to the tangle formation due to Aβ. Some studies have suggested that when APOEε4 is not lipid-bound, it binds to Aβ more strongly than other variants, but when lipid-bound, it binds to Aβ less than other APOE variants [27]. In addition, complete lipidation has been shown to affect receptor access [121]. Another interesting finding is that APOEε4 is a risk factor for Americans but not for Africans [122]. This finding, attributed to the differences in dietary habits and lipid intake and accumulation in the bodies of these two groups, suggests that APOEε4, when saturated with lipid, increases the risk of elevated tau phosphorylation and even Aβ deposition.

**Figure 4:**
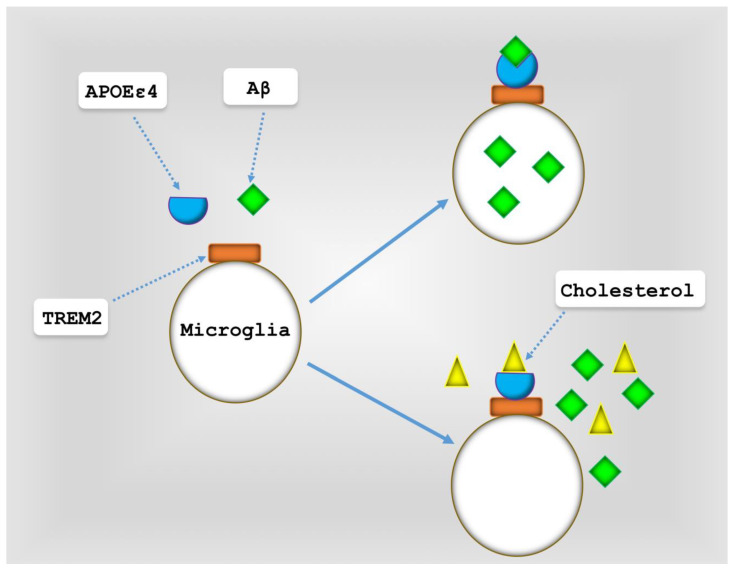
The impact of cholesterol on APOEε4 function. APOEε4 strongly impairs Aβ clearance in the presence of cholesterol.

The molecular network of the immune system and microglia is associated with late-onset AD. A number of these proteins are involved in Aβ clearance, and decreased Aβ clearance has been observed in AD [102]. As individuals age, the morphology and function of microglia change [123], and microglia dysfunction has been diagnosed in AD [124]. The number of senescent microglia increases with age, but to a much greater in AD [125]. It has been shown that the degenerative structure of tau is more associated with senescent microglia rather than active microglia. Furthermore, Aβ deposition was not found to be associated with active microglia [85]. Microglia exhibit various subpopulations in aging and AD, including neuroprotective and neurodegenerative (senescent) types. The transition between these two phenotypes is influenced by APOE signaling induced by TREM2 [102]. As discussed in the present study, APOE plays a role in Aβ clearance through TREM2. APOEε4 in the presence of lipids and cholesterol severely disrupts Aβ clearance (Figure 4). TREM2 itself has two variants, R47H and R62H, which are risk factors for AD and have been shown to reduce the internalization of Aβ monomers and decrease Aβ degradation within microglia [102]. Additionally, factors like diabetes and obesity, which are AD risk factors, have been demonstrated to convert microglia into a neurodegenerative state [126].

One more risk factor to consider is cholesterol. Elevated cholesterol levels can increase the chances of lipid raft formation, where Aβ is produced. This leads to the production of Aβ and its dimers, which can create channels in the neuron membrane, allowing calcium to enter and triggering the formation of NFTs. Furthermore, consuming cholesterol along with copper has been linked to a higher risk of AD. Copper can lead to the oxidation of cholesterol, which can then pass through the BBB [127], especially in older individuals when the BBB may not function as effectively. Additionally, an excess of cholesterol in the brain can impede Aβ clearance by APOEε4 and microglia.

Additionally, the impact of plaques on the rise of tangles can be elucidated by this hypothesis [11,12]. As a plaque develops, the level of soluble Aβ-dimers elevates, resulting in a greater likelihood of channel formation in the neuronal membrane, consequently promoting tangle formation.

An additional topic that can be explained by this hypothesis is the higher prevalence of AD in developed countries. Several factors render these nations more susceptible to AD. Longer life expectancy leads to a rise in the population of individuals with the main risk factor (the elderly). Moreover, greater hygiene [128] and copper plumbing in these countries eradicate numerous germ species while fostering the growth of resistant germs. Additionally, obesity (due to more food intake and less mobility because of greater welfare) may impact the APOEε4 function.

According to the present hypothesis, the cause of neurofibrillary tangle-predominant dementia (NFTD) can also be explained. When microglia age to the point where their ability to engulf and digest is severely diminished, it leads to the accumulation of Aβ dimers in the synaptic space, as well as an upsurge in calcium channels. The excessive number of channels results in an influx of calcium into the neuron, leading to the formation of the overabundance of tangles, both of which can lead to neuronal death and the manifestation of dementia symptoms due to substantial neuron loss.

An additional concern that aligns perfectly with the present hypothesis is the increased prevalence of AD in women. As per this hypothesis, tangles tend to form earlier and more prominently in brain areas with heightened activity, as these areas exhibit elevated Aβ production and clearance [28]. Conversely, research indicated that women’s brains are more active than men’s, especially in specific regions [129], resulting in higher Aβs production in these regions. Consequently, the likelihood of Aβ dimer formation, subsequent calcium channel activation, tau phosphorylation, and ultimately neuron death and dementia is greater in these regions. A recent study has revealed that women exhibit higher Aβ accumulation in their brains than men [130], supporting the notion of increased Aβ production in women’s brains. Furthermore, certain reports have highlighted that female mouse models of AD are more susceptible to microglia dysfunction in adulthood compared to male mice [131]. Further investigation on AD mouse models has demonstrated that aged female microglia lose their ability to modulate their phagocytic activity under inflammatory conditions [132]. Some studies have also suggested a stronger correlation between APOEε4 and tau in women, with women in the entorhinal region exhibiting more tau-mediated metabolic dysfunction [133].

## 7. Seemingly Challenging Groups for the Hypothesis

The tangle threshold can manifest in a brain that is approximately seventy years old, but two groups of young individuals with AD appear to challenge this notion: those with Down syndrome and early-onset AD. However, all available evidence indicates that these two groups not only do not defy this rule but also validate its accuracy. Patients with Down syndrome exhibit either complete or partial trisomy of chromosome 21 (which carries APP and some other AD-related genes). Even though the chromosome fragment implicated in Down syndrome does not contain any known genes [134]. A significant proportion of patients with Down syndrome develop AD after the age of 40 [135]. It is noteworthy that most of these individuals experience premature aging and their physiological age is much older than their chronological age [136]. An interesting study in this area [137] revealed that out of two individuals with Down syndrome, approximately 46 and 48 years old, possessing the same APOE genotype (e3/e3) and Aβ load, one had AD and the other did not. The affected individual carried NFTs and senile plaques, whereas the unaffected individual did not. A notable finding was the result of their autopsy, which revealed that the affected individual was 10–20 years older than his chronological age. In this scenario, genetic or physiological changes have elevated an individual’s biological age to approximately seventy. However, in patients with Down syndrome, despite having a high APP and Aβ load, AD does not manifest in children or adolescents, and dementia typically emerges from around the fifth decade onwards [4]. The spread pattern of tau pathology in patients with Down syndrome mirrors that of AD patients [138].

Early-onset AD patients present another challenge to the validity of the hypothesis that tangles reach the threshold in a nearly 70-year-old brain. Braak’s table indicates that individuals with early-onset AD had high-intensity tangles at a young age, even as early as 47 years old [7]. This suggests that genetic mutations have caused changes in their bodies, accelerating the process of reaching the threshold. As a result, tangles are observed to reach the threshold after approximately five decades. It is important to note that no child or young person was reported to be infected in this group.

## 8. Alzheimer’s Disease in Animals

In the field of AD, the longer lifespan of humans compared to animals distinguishes them, even if amyloid production and brain activity levels are similar. The absence of AD in animals further supports the presented hypothesis, as animals do not live as long as humans (except in a few cases such as parrots where dementia information is not available). Tangles reach the required threshold level after about seven decades of life, a phenomenon not observed in animals such as mice due to their shorter lifespan. In mutant mice that produce Aβ, tau is not produced, and neuronal loss does not occur [139] because tangles have not provided the necessary background before the Aβ increased; in addition, the mice brains were not exposed to infectious agents that provided similar conditions to AD in humans. Even in monkeys, which are models closer to humans, tangles are not seen [139] because they have a shorter lifespan than humans. For example, when comparing rhesus and marmosets, which have lifespans of about 16 and 35 years, respectively, to humans who live approximately 80 years, the hypothesis proposed in this study implies that the brain may not have the necessary conditions for tangle accumulation.

## 9. Conclusions

Based on the contents of the present study, it can be inferred that as microglia age and progress into a dysfunctional state characterized by impaired phagocytosis and heightened production of proinflammatory cytokines, the clearance of Aβ is compromised. The accumulation of excess Aβ can result in increased calcium influx into neurons through the formation of channels, subsequently leading to tau phosphorylation and the formation of tau tangles. As microglia age and their functionality decreases, the clearance of germ debris becomes inefficient, leading to the formation of diffuse plaques. In cases where resistant microbes enter sensitive brain regions that utilize high copper levels to inhibit NMDA receptors, the affected neurons are destroyed, resulting in the formation of senile plaques. The manifestation of AD symptoms occurs when the infection or neuronal loss is significant.
